# Conservation genetics and phylogeography of endangered and endemic shrub *Tetraena mongolica *(Zygophyllaceae) in Inner Mongolia, China

**DOI:** 10.1186/1471-2156-12-1

**Published:** 2011-01-04

**Authors:** Xue-Jun Ge, Chi-Chuan Hwang, Zin-Huang Liu, Chi-Chun Huang, Wei-Hsiang Huang, Kuo-Hsiang Hung, Wei-Kuang Wang, Tzen-Yuh Chiang

**Affiliations:** 1 Key Laboratory of Plant Resources Conservation and Sustainable Utilization, South China Botanical Garden, Chinese Academy of Sciences, Guangzhou 510650, PR China; 2 Department of Engineering Science, National Cheng-Kung University, Tainan 701, Taiwan; 3 Department of Biological Sciences, National Sun Yat-Sen University, Kaohsiung 804, Taiwan; 4 Department of Life Sciences, National Cheng-Kung University, Tainan 701, Taiwan; 5 Graduate Institute of Bioresources, Pingtung University of Science and Technology, Pingtung 912, Taiwan

## Abstract

**Background:**

*Tetraena mongolica *(Zygophyllaceae), an endangered endemic species in western Inner Mongolia, China. For endemic species with a limited geographical range and declining populations, historical patterns of demography and hierarchical genetic structure are important for determining population structure, and also provide information for developing effective and sustainable management plans. In this study, we assess genetic variation, population structure, and phylogeography of *T. mongolica *from eight populations. Furthermore, we evaluate the conservation and management units to provide the information for conservation.

**Results:**

Sequence variation and spatial apportionment of the *atp*B-*rbc*L noncoding spacer region of the chloroplast DNA were used to reconstruct the phylogeography of *T. mongolica*. A total of 880 bp was sequenced from eight extant populations throughout the whole range of its distribution. At the cpDNA locus, high levels of genetic differentiation among populations and low levels of genetic variation within populations were detected, indicating that most seed dispersal was restricted within populations.

**Conclusions:**

Demographic fluctuations, which led to random losses of genetic polymorphisms from populations, due to frequent flooding of the Yellow River and human disturbance were indicated by the analysis of BEAST skyline plot. Nested clade analysis revealed that restricted gene flow with isolation by distance plus occasional long distance dispersal is the main evolutionary factor affecting the phylogeography and population structure of *T*. *mongolica*. For setting a conservation management plan, each population of *T*. *mongolica *should be recognized as a conservation unit.

## Background

Genetic variation within and among natural populations is crucial for the long-term survival of a species. An accurate estimate of the level and distribution of genetic diversity of threatened species provides fundamental information in designing conservation programs [1,2]. *Tetraena mongolica *Maxim, a monotypic genus of the Zygophyllaceae, is endemic to the western part of Inner Mongolia around the basin of the Yellow River [3], and is also subjected as nationally endangered in China [4]. Plants of *T. mongolica*, up to 0.5 m in height, flower from mid-May till early June, and set fruits in July. The species is restrictedly distributed in the western Gobi, the largest desert in Asia characterized by extreme low annual rainfall [3], where *T. mongolica *with a fully developed root system is well adapted and becomes locally dominant. *T. mongolica *plays an important ecological role as windbreak for stabilizing river bank [5]. Nevertheless, it has been used as firewood, locally called as "oil firewood" because its stems are combustible even in fresh state due to containing high levels of triacylglycerol [6]. Human's overexploitation has inevitably caused a dramatic decline of the species.

Previous studies have been focusing on the biological characters causing the population decline of *T. mongolica*. As the high rate of ovule abortion after anthesis [7], the seed-set of *T. mongolica *was quite low (1.3 - 2.8%) in the natural populations [8,9]. Previous population genetic researches based on allozyme and ISSR data revealed medium levels of genetic differentiation among populations of *T. mongolica *[3,10].

Understanding levels and spatial partitioning of genetic polymorphisms in an endangered species provides sufficient information for conservation practices. This kind of researches has become increasingly popular in the recent years, with the development of analytical methods to take phylogenetic distinctiveness into account when setting conservation priorities [11,12]. During the past few decades, the theoretical framework of population genetics and empirical data gathered with the help of molecular genetic methods have been widely used in conservation biology [13]. Given a haploid nature and a low frequency of genetic recombination, molecular markers of organelle DNA have been long used for phylogenetic reconstruction at various taxonomic levels, conservation genetics, and assessing the migratory routes of species [14,15]. Although chloroplast DNA evolves relatively slowly, moderate to high levels of genetic variation have frequently been detected in some noncoding spacers within and among species [16-18]. With maternal inheritance [19], cpDNA is suitable for investigating processes associated with seed dispersal, such as range expansions [20] and the contribution of seed movement to total gene flow [21,22].

For endemic species with a limited geographical range and declining populations, historical patterns of demography and hierarchical genetic structure are important for determining population structure, and also provide information for developing effective and sustainable management plans [23]. In this study, we investigated genetic variation, population structure, and phylogeography of *T. mongolica *from eight populations throughout the entire distribution range. Several aims are pursued: 1) to examine the levels of genetic variation within and between populations, 2) to reconstruct phylogeographical patterns and examine the extent of genetic differentiation among populations, and 3) to identify the conservation and management units based on genetic evidence, to provide the information for the development of effective and efficient conservation practices for this species.

## Results

### Genetic diversity and cpDNA phylogeny of *T. mongolica*

No within-individual variation was detected in the non-coding spacer between *atp*B and *rbc*L genes of the chloroplast DNA. Identical sequences were obtained from five clones derived from the same amplification reaction, indicating no PCR artifacts caused by *Taq *polymerase or sequencing errors. The *atp*B-*rbc*L intergenic region of cpDNA in *T. mongolica *varied from 872 to 880 base pairs (bp) in length. The cpDNA sequences were aligned with a consensus length of 881 bp, of which 46 sites (5.2%) were variable.

**Figure 1:**
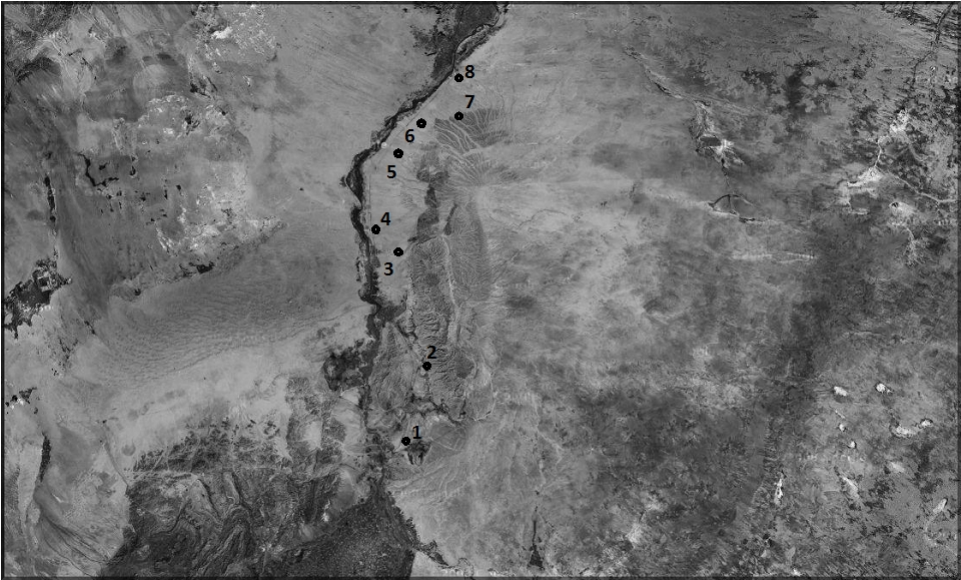
**Map showing population locations of *Tetraena mongolica *sampled**. Population names are 1. Shizuishan, 2. Hainanqu, 3. Qianlishan, 4. Xindi, 5. Yikebulage, 6. Taosita, 7. Muoshigou, 8. Balagong.

The chloroplast spacer is A/T rich with an average content of 73.6%, which is consistent with the nucleotide composition of most noncoding spacers and pseudogenes because of low functional constraints [24]. In total, 38 haplotypes (GenBank accession numbers of HQ142910-HQ142986) were identified from 77 individuals of *T. mongolica*, with an estimated haplotype diversity of *h *= 0.962 ± 0.009 (Table 1; Figure 1). Except for the monomorphic population of YKBLG, haplotype diversity varied across populations, ranging between 0.378 (GLS) and 1.000 (HN). Low levels of nucleotide difference were detected within the whole species (*θ *= 0.00447 ± 0.0003) and within populations, ranging from *θ *= 0.00099 (TST) to 0.00405 (HN).

**Table 1:** Population locations, numbers of sample size and site coordinate of Tetraena mongolica, the estimates of haplotype diversity (h) and nucleotide difference (θ) within populations on cpDNA sequences.

Population	Code	Site coordinate	Elevation	Sample size (N)	Number of haplotypes	Polymorphic sites (S)	* h *± SD	* θ *± SD
1. Shizuishan	SZS	106°49'E 39°25'N	1130 m	10	4	4	0.733 ± 0.120	0.00146 ± 0.00037
2. Hainanqu	HN	106°54'E 39°33'N	1280 m	10	10	9	1.000 ± 0.045	0.00405 ± 0.00041
3. Qianlishan	GLS	106°50'E 39°50'N	1170 m	10	3	7	0.378 ± 0.181	0.00160 ± 0.00105
4. Xindi	XD	106°46'E 39°52'N	1090 m	10	7	9	0.933 ± 0.062	0.00334 ± 0.00069
5. Yikebulage	YKBLG	106°49'E 40°05'N	1070 m	8	1	0	0.000 ± 0.000	0.00000 ± 0.00000
6. Taositu	TST	106°54'E 40°09'N	1070 m	10	4	3	0.644 ± 0.152	0.00099 ± 0.00030
7. Muoshigou	MSG	107°04'E 40°07'N	1380 m	10	4	3	0.733 ± 0.101	0.00110 ± 0.00025
8. Balagong	BLG	107°03'E 40°16'N	1100 m	9	6	9	0.833 ± 0.016	0.00249 ± 0.00066

Overall				77	38	44	0.962 ± 0.009	0.00447 ± 0.00030

**Figure 2:**
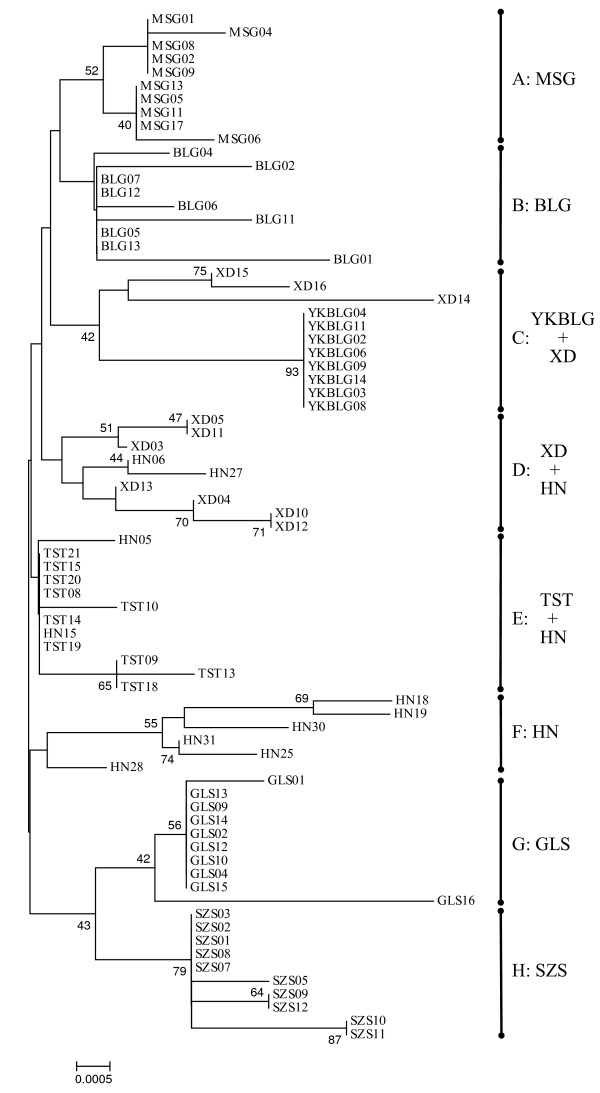
**Neighbor-joining tree of *Tetraena mongolica *based on sequences of the *atp*B-*rbc*L intergenic spacer of cpDNA**. Numbers at nodes are bootstrap values. See Table 1 for the acronyms of population names.

A neighbor-joining tree obtained using MEGA recovered eight cpDNA clades (Figure 2). Most of the populations contained only one clade in the genetic composition, except for the populations XD and HN. Apparently, most genetic variation resides between populations of *T. mongolica*.

### Nested clade analysis, phylogeography and population differentiation

**Figure 3:**
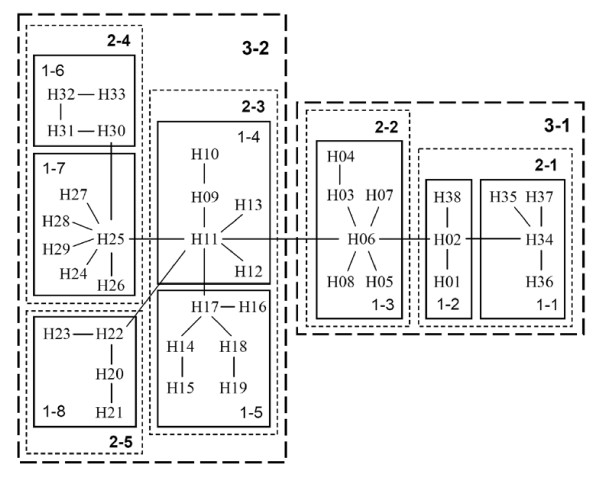
**Minimum-spanning network based on mutations between haplotypes of the *atp*B-*rbc*L noncoding spacer of cpDNA of *Tetraena mongolica***.

A nested clade analysis was accomplished by linking nucleotide haplotypes in a hierarchical manner based on mutational changes. After linking the haplotypes into a clade, closely related clades were linked further to form a higher-level group; via such hierarchical linking, a nested network was drawn (Figure 3). In total, 38 haplotypes (H1-H38) and 8 clades, 1-1 to 1-8, were identified. The distribution of haplotypes and clades in populations was indicated in Tables 2 and 3. Clades 1-1 to 1-3 were clustered into clade 2-1, which was further grouped with clade 2-2 into a higher-level clade 3-1. Likewise, clades 2-3 and 2-4, which consisted of clades 1-4 to 1-5 and 1-6 to 1-7, respectively, were clustered and nested with clade 2-5 in the higher-level clade 3-2. Clades 3-1 and 3-2 corresponding to clades G-H and A-E of the NJ tree, respectively, were identified. The former is distributed in the southern part of the distributional range, while the latter is in the northern part. The topology showing geographical division is shared by the minimum spanning network and the NJ tree. Most populations were genetically differentiated, as most haplotypes were private within populations except for H11, which were shared by populations TST and HN (Table 2).

**Table 2:** Individual number and geographical distribution of haplotypes in populations of Tetraena mongolica based on cpDNA data.

	SZS	HN	GLS	XD	YKBLG	TST	MSG	BLG	Total
H01			1						1
H02			8						8
H03		1							1
H04		1							1
H05		1							1
H06		1							1
H07		1							1
H08		1							1
H09						1			1
H10						2			2
H11		1				6			7
H12						1			1
H13		1							1
H14		1							1
H15		1							1
H16				2					2
H17				2					2
H18				2					2
H19				1					1
H20							4		4
H21							1		1
H22							4		4
H23							1		1
H24								1	1
H25								4	4
H26								1	1
H27								1	1
H28								1	1
H29								1	1
H30					8				8
H31				1					1
H32				1					1
H33				1					1
H34	5								5
H35	1								1
H36	2								2
H37	2								2
H38			1						1

Total	10	10	10	10	8	10	10	9	77

**Figure 4:**
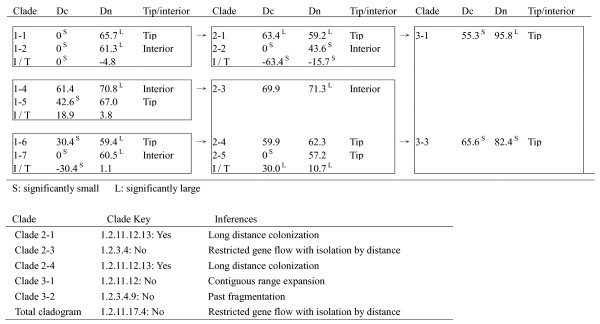
**Result of the nested clade analysis**. Clade (*Dc*) and nesting clade (*Dn*) distances are given for each level of the nesting design. *Superscripts *refer to significantly small (*S*) or large (*L*) clade and nested clade distances. Inferences of current population structure and population history based on nested clade analysis and the interpretation key given in Templeton et al. (1995) are indicated at the bottom of the figure.

A nested contingency analysis detected significant geographical associations within several clades (2-1, 2-3, 2-4, 3-1 and 3-2) and the whole cladogram. The phylogeographical inferences are listed in Figure 4. Most tip-clades were restricted to unique regions, whereas basal interior clade 1-4 was widespread (Table 3, Figure 3). The results agree with the hypothesis of constrained seed dispersal of the species. The deduced *Nm *of 0.04-0.71 and *F*_*ST *_of 0.38-0.90 indicated high levels of genetic differentiation between all populations, with three exceptional pairs of HN-TST, HN-XD and XD-TST (Table 4). An "isolation by distance" model across eight populations of the species was supported by a regression test between *Nm *values and geographical distance (R = 0.772, P < 0.05).

**Table 3:** Number of individuals and geographical distribution of clades in populations of Tetraena mongolica based on cpDNA data.

Clade^a^	Individual (*n*)	SZS	HN	GLS	XD	YKBLG	TST	MSG	BLG
1-1	10	10							
1-2	10			10					
2-1	20 (26.0%)	10		10					
1-3	6		6						
2-2	6 (7.8%)		6						
3-1	26 (33.8%)	10	6	10					
1-4	12		2				10		
1-5	9		2		7				
2-3	21 (27.3%)		4		7		10		
1-6	11				3	8			
1-7	9								9
2-4	20 (25.9%)				3	8			9
1-8	10							10	
2-5	10 (13.0%)							10	
3-2	51 (66.2%)		4		10	8	10	10	9

Total	77	10	10	10	10	8	10	10	9

**Table 4:** Pairwise comparisons of deduced Nm (below diagonal) and Fst (above diagonal) between populations of Tetraena mongolica based on cpDNA sequences.

	SZS	HN	GLS	XD	YKBLG	TST	MSG	BLG
SZS		0.54	0.64	0.56	0.90	0.70	0.78	0.65
HN	0.42		0.50	0.20	0.66	0.26	0.41	0.37
GLS	0.28	0.50		0.65	0.86	0.65	0.73	0.60
XD	0.39	2.01	0.44		0.68	0.27	0.47	0.38
YKBLG	0.06	0.26	0.04	0.24		0.86	0.85	0.68
TST	0.21	1.44	0.27	1.36	0.08		0.59	0.40
MSG	0.07	0.71	0.19	0.28	0.04	0.18		0.45
BLG	0.13	0.68	0.33	0.41	0.12	0.37	0.30	

Relative values of *Dc *and *Dn *for each clade representing contemporary distributions of haplotypes were used to interpret historical and contemporary gene flow processes (Figure 4). Restricted gene flow with isolation by distance was the primary process responsible for the present-day distribution in Inner Mongolia (total cladogram), also inferred for clade 2-3. While some other different inferences, like long distance colonization and past fragmentation were detected at clade clades 3-1 and 3-2, respectively. This result shows limited seed dispersal of this species, while with occasional long distance dispersal [25].

### Population demography pattern of *T. mongolica*

**Figure 5:**
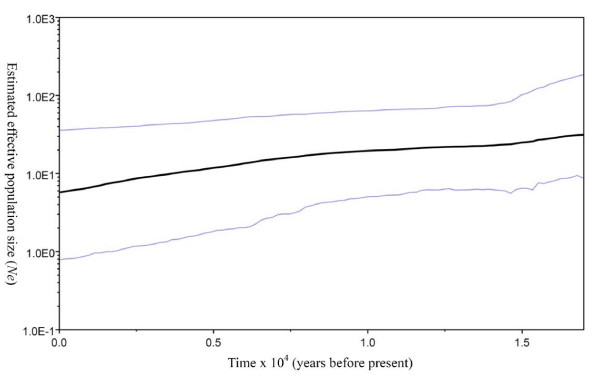
**Median effective population size (thin lines indicate 95% highest posterior density interval) as estimated from cpDNA *atp*B-*rbc*L spacer sequence data in program BEAST using a Bayesian skyline plot technique**.

Historical population dynamics of *T. mongolica *was estimated using Bayesian skyline plots, a coalescent Markov chain Monte Carlo method that does not require an assumed parametric model of demographic history. The skyline diagrams, which summarize instantaneous estimates of effective population size, showed recent population decline for *T. mongolica *over the last sixteen thousand years (Figure 5). The shape of the skyline diagram conforms to its known history. That is, habitat destruction and misapplication of human activity and the frequent flooding of the Yellow River may have caused a decline in *T. mongolica *population size.

## Discussion

### Genetic variation of the *atp*B-*rbc*L noncoding spacer of cpDNA in *T. mongolica*

In this study, we investigated the phylogeographical pattern and population structure of endangered *T. mongolica *in western Inner Mongolia. In total, 38 haplotypes were detected at the cpDNA *atp*B-*rbc*L locus in *T. mongolica*. The level of genetic variation of cpDNA is comparable to that of other endangered shrub plants, e.g., *Dunnia sinensis *(*θ *= 0.0022) [26], and *Hygrophila pogoncalyx *(*θ *= 0.00343) [17], but is lower compared to other endangered species, e.g., *θ *= 0.01018 for the cpDNA *trn*D-*trn*T spacer of *Cunninghamia konishii *[27], and *θ *= 0.01268 for the cpDNA *atp*B-*rbc*L spacer of *Cycas taitungensis *[28]. The twofold lower nucleotide diversity to above endangered species in *T. mongolica *may be ascribed to its extremely small effective population size associated with the low seed set in wild (1.3 - 2.8%) [9].

Recent habitat loss has reduced the number and size of *T. mongolica *populations [10]. Small populations of narrowly distributed species are expected to exhibit low levels of genetic variation, but high levels of genetic differentiation among populations, which were all detected in this species (Table 4) [2]. Interestingly, different levels of genetic variation were detected in different populations. The HN and XD populations possessed more haplotypes and higher genetic diversity than others, whereas YKBLG population displayed genetic homogeneity (Table 1). The lack of genetic variability in some populations, e.g. SZS, MSG and TST, near threefold lower in nucleotide diversity, was likely associated with frequently human activities. In contrast, some populations of *T. mongolica *experienced relatively little disturbance due to low accessibilities [3,10].

Our previous study revealed medium levels of genetic differentiation among populations of *T. mongolica *based on ISSR data [10]. In contrast, in cpDNA spacer higher genetic differentiation was detected between populations than in ISSR fingerprinting. The difference may be highly associated with the reproductive characteristics of the species. It has been known that gene flow of seed plants occurs either via pollen prior to fertilization or seeds. In this study, *T. mongolica *is primarily pollinated by insects [29]. Gene flow between populations via pollen would be limited by the migratory capacity of pollinators. In addition, seed dispersal of seeds from schizocarp, a dry fruit developing from four carpels, is constrained by gravity [29], likely resulting in most seed dispersal confined to short distances. With maternal inheritance and haploid nature, chloroplast DNA is suitable for estimating the contribution of seed movement to total gene flow [21], whereas, ISSRs represent nuclear DNA, mostly carried and dispersed by pollen dispersal [30]. In this study, higher genetic differentiation between all populations in cpDNA than in ISSR is likely ascribed to limited seed dispersal.

The BEAST skyline plot for cpDNA spacer identified a recent population decline ever since sixteen thousand years before present likely associated with human destruction as *T. mongolica *has long been used as firewood (Figure 5) [6]. Ecologically, this plant is still one of the dominant shrubs in Inner Mongolia. Through the analysis of skyline plot, we were able to recover the history of a long term human disturbance that caused a decline in population size of *T. mongolica*.

Another major factor that shaped the phylogeography and population demography is the frequent flooding of the Yellow River, the second longest river in China [31]. The floods not only eroded river banks, but resulted in many habitats submerged, inevitably leading to population extinction. In addition to bank erosion, the Yellow River is well known for its heavy load of silt. Soil deposits elevate the riverbed and cause flows between natural levees. The river may break out of the levees into the surrounding lower flood plain and adopt a new route. Records indicate that the events have occurred about once every century [32]. Such devastations caused dramatic changes of flora and fauna along the Yellow River. Geological records indicate that the river's levees were breached more than 1,500 times and its course changed 26 times in the last 3,000 years [32]. Given such frequent flooding, *T. mongolica *would have experienced demographic fluctuations over and over. That is, severe periodical population bottlenecks followed by subsequent demographic expansion would elevate genetic drift effects and lead to a loss of genetic variation [33,34].

### Phylogeography and conservation of *T. mongolica*

In this study, gene genealogy of cpDNA in *T. mongolica *was recovered (Figures 2 and 3). Eight cpDNA clades were identified in the NJ tree. Most of the populations contained only one clade in the genetic composition, displaying a pattern of most genetic variation residing between populations. Nested contingency analysis discriminating the geographical associations of haplotypes and clades provides further insights into historical events that shaped the phylogeography (Figure 4). At the total cladogram, restricted levels of *Dc *vs. a large *Dn *illustrates restricted gene flow with isolation by distance as the primary process responsible for the present-day distribution of *T. mongolica *in Inner Mongolia. As cpDNA is maternally inherited, this inference indicates limited seed dispersal. Besides, long distance colonization was also observed in clade 3-1, a common phenomenon occurring over glacial maxima [25]. Furthermore, past fragmentation observed in clade 3-2 was likely associated with the Yellow River flooding.

It is expected that endangered species that are narrowly distributed and own a small population size would have high risks of extinction, especially when gene flow between populations is restricted [1,35]. Another consequence of a small-sized population is the susceptibility to inbreeding, which reduces heterozygosity and the performance of various fitness-related traits, thereby substantially increasing the probability of extinction [36,37]. Given small sizes in the wild populations of *T. mongolica*, the maintenance of genetic diversity would be critical for the long-term survival of species in considering conservation strategies [38]. Historical demographic events in a species play an important role in determining the present-day geographic structure of intraspecific genetic variation [39]. In this study, given the high levels of population differentiation between populations and low levels of genetic diversity within populations of *T. mongolica*, for retaining the existing diversity, reservation regions covering major populations with high genetic variation should be established.

Habitat destruction and fragmentation would inevitably result in small and isolated populations [40,41], and increase the risk of population extinction. Today, many natural habitats of *T. mongolica *around the Yellow River have been destroyed or altered due to human overexploitation. In order to develop an effective strategy to conserve species, the Evolutionarily Significant Unit (ESU) needs to be defined. Various criteria for ESUs have been suggested, including reciprocal monophyly [23], adaptive variation [42], and reproductive separation [43]. Recognizing ESUs as reciprocally monophyletic groups ensures that the entire evolutionary heritage within species can be maintained and that populations belonging to different lineages can be managed separately [44]. In *T. mongolica*, given high levels of genetic differentiation and reciprocal monophyly between most populations and lower genetic variation within populations, each population should be treated as different evolutionarily significant units for conservation (Figure 2).

From another view, conservation efforts can be targeted to genetic hot spots where populations have high levels of genetic diversity [17,45]. Accordingly, HN and XD populations that owned higher genetic diversity near two-three folds than other populations (Table 1) can be recognized as genetic hot spots of *T. mongolica*. The concept of genetic and ecological exchangeabilities is also central to the definitions of ESU [46]. Crandall et al. [44] emphasize that the ESU concept not only includes ecological data and genetic variation, but also considers the ecological and genetic exchangeabilities. In practice, the status of recent and historical genetic and ecological exchangeability between populations is considered. As the contemporary gene flow from the populations at genetic hot spots to other populations does not exist or cannot be determined, the populations at the genetic hot spots need to be treated as distinct conservation units for its unique genetic variation [44]. Accordingly, hotspots at HN and XD of *T. mongolica *would represent different units for conservation, as seed dispersal between populations is limited.

## Conclusion

As expected, the rare species of *T. mongolica *possessed very low genetic variation at the cpDNA noncoding spacer. The effects of random genetic drift would lead to the loss of genetic variability in a small-sized population and genetic differentiation among populations, as observed in *T. mongolica *that has been mediated by limited seed dispersal and frequent population shrinking due to the flooding of the Yellow River. Phylogeographical analyses suggest that the present-day *T. mongolica *populations in Inner Mongolia were likely a result of restricted gene flow with isolation by distance plus occasional long distance dispersal. Molecular data provide sufficient information for reconstructing phylogeographical patterns and critical information for setting conservation strategy. For conservation considerations we suggest that populations with reciprocal monophyly at the cpDNA, and the two genetically highly variable populations likely represent management units for protecting *T. mongolica*.

## Methods

### Population sampling

In this study, we investigated the genetic variation and structure in the rare and endangered *T. mongolica*. This rare species is distributed in the western Inner Mongolia with small habitat fragments. Samples were collected from all populations, avoiding clonal individuals. A total of 77 individuals, representing eight extant populations, were sampled throughout the species entire range (Figure 1, Table 1). Individuals were chosen randomly. Young healthy leaves were collected from the field and were dried directly with silica gel. Dried leaves were preserved in silica gel until DNA extraction.

### DNA extraction and PCR

Leaves were powdered in liquid nitrogen and stored in a -70°C freezer. Genomic DNAs were extracted from the powdered tissue following a CTAB procedure and gel-quantified [47]. The intergenic spacer between the *atp*B and *rbc*L genes of the chloroplast DNA was amplified using a pair of universal primers [48]. Each 50 μL PCR contained: 10 ng template DNA, 5 μl 10 × PCR reaction buffer, 5 μL MgCl_2 _(25 mM), 5 μl dNTP mix (8 mM), 10 pmole of each primer, and 4 U of *Taq *polymerase (Promega, Madison, USA). The reaction was programmed on an MJ Thermal Cycler with first cycle of denaturation at 95°C for 2 min, then 30 cycles of denaturation at 94°C for 45 sec, annealing at 48°C for 1 min, and extension at 72°C for 1 min 30 sec, followed by 72°C extension for 10 min and 4°C for storage.

### T-vector cloning and nucleotide sequencing

All PCR products were purified from an agarose gel using the PCR product purification kit (Viogene, Sunnyvale, USA) and cloned into a pGEM-T easy cloning vector (Promega). For each cpDNA *atp*B-*rbc*L fragment, both strands were cycle-sequenced using the *Taq *Dye Deoxy Terminator Cycle Sequencing Kit (Applied Biosystem, Foster City, USA). Products of the cycle sequencing reactions were run on an ABI 377XL automated sequencer (Applied Biosystem). Cloned PCR products were sequenced using universal T7 forward (5'- TAATACGACTCACTATACGGG-3') and SP6 reverse (5'-TATTTAGGTGACACTATAG-3') primers located on pGEM-T easy vector termination sites. Five clones for each individual were randomly chosen and sequenced.

### Sequence alignment and phylogenetic analysis

The chloroplast DNA sequences were aligned with the program Clustal X 1.81 [49]. Indels were excluded from the data analysis. Neighbor-joining (NJ) analysis based on Kimura's [50] two-parameter distance was performed using the software MEGA 4.0 [51]. To evaluate clade support, 1,000 replicates of bootstrap analysis [52] were performed using fast heuristic search and TBR branch-swapping. The nested clade analysis of Templeton et al. [53] provides a statistical framework for examining associations between the geographical distribution of haplotypes and their genealogical relationships [54,55]. Pairwise comparisons between DNA haplotypes were calculated using MEGA 4.0 [51]. These were used to construct a minimum spanning network in a hierarchical manner with the aid of the MINSPNET [56].

The nested clade analysis was accomplished by linking nucleotide haplotypes in a hierarchical manner based on mutational changes. The linking rules start at the tips of the cladogram and move one mutational step into the interior, uniting all haplotypes connected by this procedure into a '1-step clade'. Following pruning off the initial 1-step clades from the tips, the procedure is repeated on the more interior portions of the haplotype tree until all haplotypes have been placed into 1-step clades. The next level of nesting uses the 1-step clades as units, rather than individual haplotypes. The linking rules are the same; however, '2-step clades' are now formed. The nesting procedure is repeated until a nesting level is reached such that the next higher nesting level would result in only a single category spanning the entire original haplotype network; via such hierarchical linking, a nested network was drawn.

### Population genetic analysis of the cpDNA

Levels of inter- and intra-population genetic diversity based on cpDNA were quantified by indices of haplotype diversity (*h*) [57] and estimates of nucleotide difference (*θ*) [58] using DnaSP (Version 5) [59]. Patterns of geographical subdivision and gene flow were also estimated hierarchically with the aid of DnaSP. Gene flow within and among regions or populations was approximated as *Nm*, the number of female migrants per generation between populations. *Nm *was estimated using the expression F_ST _= 1/(1 *+ *2 *Nm*), where *N *is the female effective population size and *m *is the female migration rate [60].

A model of "isolation by distance" was assessed by plotting pairwise *Nm *values against geographical distance [60]. The correlation between *Nm *and distance was determined by a regression of F-test over distances using SPSS program version 6.0 [61]. Geographical associations of haplotypes and clades within the minimum spanning network were tested using the program GeoDis [62]. Two statistics were calculated: 1) the clade distance, *Dc*, a measure of the geographical spread of a clade, and 2) the nested clade distance, *Dn*, a measure of the geographical distribution of a clade relative to other clades in the same higher level nesting category. These measures of geographical distribution were used to infer historical processes following the methods of Templeton et al. [53]. The nested clade analysis (NCA) has been recently challenged with serious flaws, such as ignorance of the type I error [63]. The debates [64-66] have been lasting. In this study, we used this analysis to generate a phylogeography hypothesis.

To estimate the population demographic trends for *T. mongolica*, we analyzed the cpDNA noncoding spacer data with BEAST v1.4 [67]. Bayesian skyline plot model was used to infer past demographic dynamics through time, which uses standard Markov chain Monte Carlo (MCMC) sampling procedures calculated from a sample of molecular sequences estimate a posterior distribution of effective population size without dependence on a pre-specified parametric model of demographic history [68]. In the study, an evolutionary rate for the chloroplast *atpB*-*rbcL *spacer of 3 × 10^-9 ^substitutions per site per year was used [69,70]. The Bayesian skyline plot includes credibility intervals for the estimated effective population size at every point in time, back to the most recent common ancestor of the gene sequences. The credibility intervals represent both phylogenetic and coalescent uncertainty.

## Authors' contributions

TYC designed and conceived this study. XJG collected the samples and analyzed the data. WKW, ZHL, CCH, KHH and WHH collected the genetic data. WKW and TYC analyzed the data and wrote the manuscript. All authors read and approved the final manuscript.
